# The impact of restricting US cigarette sales to state-controlled outlets on tobacco-related mortality

**DOI:** 10.1093/ntr/ntag084

**Published:** 2026-04-21

**Authors:** Sarah D Mills, Jiaqian Fan, Megan C Diaz, Shelley D Golden, Christopher A Wiesen, Jidong Huang, Kurt M Ribisl

**Affiliations:** Department of Health Behavior, Gillings School of Global Public Health, University of North Carolina at Chapel Hill, Chapel Hill, NC, United States; Lineberger Comprehensive Cancer Center, University of North Carolina at Chapel Hill, Chapel Hill, NC, United States; Department of Biostatistics, Gillings School of Global Public Health, University of North Carolina at Chapel Hill, Chapel Hill, NC, United States; Schroeder Institute, Truth Initiative, Washington, DC, United States; Department of Health Behavior, Gillings School of Global Public Health, University of North Carolina at Chapel Hill, Chapel Hill, NC, United States; Lineberger Comprehensive Cancer Center, University of North Carolina at Chapel Hill, Chapel Hill, NC, United States; Odum Institute, University of North Carolina at Chapel Hill, Chapel Hill, NC, United States; Department of Health Policy & Behavioral Sciences, School of Public Health, Georgia State University, Atlanta, GA, United States; Department of Health Behavior, Gillings School of Global Public Health, University of North Carolina at Chapel Hill, Chapel Hill, NC, United States; Lineberger Comprehensive Cancer Center, University of North Carolina at Chapel Hill, Chapel Hill, NC, United States

**Keywords:** policy, public health, tobacco control

## Abstract

**Introduction:**

Smoking rates have declined markedly over the past several decades prompting discussion about new strategies to reach an “endgame” in tobacco control. An innovative endgame strategy is restricting cigarette sales to state-controlled outlets to reduce availability of cigarettes. In this study we estimate the impact of restricting cigarette sales to state-controlled outlets on tobacco-related mortality in 4 US states, Oregon, Pennsylvania, Vermont, and Virginia.

**Methods:**

In each state, we used Monte Carlo simulation with repeated random sampling to estimate the number of reduced deaths over a 20-year period due to reduced cigarette consumption after cigarette sales are restricted to state-controlled outlets. Model estimates were informed by data on the number of annual deaths due to cigarette smoking from the Global Burden of Disease Study and estimates about sales, a proxy for consumption, and mortality risk from the literature.

**Results:**

We estimated that over a 20-year period 366 (95% uncertainty interval [UI]: 169, 652), 1585 (95% UI: 735, 2773), 74 (95% UI: 34, 127), and 1064 (95% UI: 501, 1806) deaths due to cigarette smoking would be avoided in Oregon, Pennsylvania, Vermont, and Virginia, respectively.

**Conclusions:**

Transitioning cigarette sales to state-controlled outlets appears to be a potent tobacco control strategy that may reduce tobacco-related mortality. Policy impact on mortality may be even greater if cigarette smoking prevalence is reduced more than we estimated. To reach an endgame, states should prioritize more aggressive tobacco control policies that limit the availability of cigarettes.

**Implications:**

Findings from this study suggest that transitioning cigarette sales to state-controlled outlets is a strong tobacco control strategy that may reduce tobacco-related mortality. We found that in each of the 4 US states evaluated the policy would reduce cigarette-related deaths over a 20-year period.

As smoking rates reach historic lows in the United States (US), discussions in tobacco control have turned to how to reach an “endgame” with smoking rates below 5% in the adult US population.^1^ Incremental tobacco control measures are unlikely on their own to eliminate the remaining burden of tobacco use in the US. More aggressive approaches that transform commercial availability have been proposed, including tobacco control strategies focused on restricting the sale of cigarettes.^2^

An increasing number of cities and states implement policies that limit where tobacco products can be sold.^3,4^ For example, in 2015 San Francisco, California, capped the number of tobacco retailers permitted in each of the 11 city districts to 45 retailers.^5^ In 2021, Beverly Hills and Manhattan Beach, California, banned the sale of tobacco products altogether.^6^ Studies provide strong evidence that policies that reduce the concentration of tobacco retailers are associated with lower tobacco use among youth and adults.^7–9^

One endgame tobacco control strategy that has received less attention is restricting sales of cigarettes to state-controlled outlets. In a manner similar to states that regulate the sale of alcoholic beverages, tobacco companies would continue to manufacture cigarettes, but state and local governments would control how and where they are sold. For example, states could set prices for cigarettes and limit the types of stores that can sell them. To reduce cigarette use prevalence rates, states could increase the price of cigarettes over time, which studies show may lead to reduced consumption.^10,11^ Restricting the sales of cigarettes to state-controlled outlets would likely also reduce the concentration of tobacco retailers, which could increase the travel time and related cost associated with purchasing cigarettes, and in turn, further reduce consumption.^12^ Presently, cigarettes are readily available because they are sold in a variety of stores including convenience stores, grocery stores, gas stations, and warehouse and discount stores.^13,14^ Unlike other potential endgame policies, such as steep cigarette tax increases, restricting cigarette sales to state-controlled outlets may be a more feasible strategy, as several states already restrict alcohol sales in this manner.^15^

The present study estimates the impact of restricting cigarette sales to state-controlled outlets on tobacco-related mortality. We use simulation modeling to evaluate a hypothetical scenario where cigarette sales are restricted to state-controlled outlets in 4 US states (Oregon, Pennsylvania, Vermont, and Virginia) with varied policy and demographic contexts. We hypothesize that restricting cigarette sales to state-controlled outlets will reduce cigarette use, and in turn, lower tobacco-related mortality in each state. Findings from this study may help inform policymakers considering endgame strategies.

## Methods

Following an analytic approach similar to that used by Rostron et al. who estimated the impact of a flavored cigar ban on tobacco-related mortality,^16^ we assess the impact that restricting cigarette sales to state-controlled outlets would have on cigarette-related mortality over a 20-year period in each state by estimating the number of reduced deaths due to reduced cigarette consumption. First, in each state we use estimates of the number of annual deaths due to cigarette smoking from the Global Burden of Disease Study.^17^ Next, to inform model parameters about the reduction in cigarette consumption due to the policy, we used estimates from Diaz et al.,^12^ who estimated the policy’s impact on cigarette sales in each state, with the assumption that sales approximate consumption. They modeled 3 plausible price scenarios that would accompany the restriction of sales to state-controlled outlets: (1) states maintain cigarette pack prices at their current price, (2) states increase cigarette pack prices by 7%, or (3) states increase cigarette pack prices to meet a consumption target reduction of 5%.^12^ Using a linear price model based on the total own-price elasticity of demand, they estimate how both direct and indirect price increases as a result of the policy would affect cigarette pack sales.^12^ Each model accounts for the direct effect of price increases as well as the indirect price effect of higher consumer travel and time costs resulting from reduced outlet density. Diaz et al.’s models incorporated a price elasticity of demand of -0.4, as recommended by the Community Preventive Service Task Force,^18^ as well as pre-policy prices and locations of tobacco retailers that were state-specific. For a detailed description of the methods used to estimate the linear pricing models, see Diaz et al.^12^

For this study, we used Diaz et al.’s^12^ estimates of state-specific changes in cigarette sales across the 3 price scenarios modeled to develop plausible ranges of policy impact. Specifically, the primary point estimate of policy impact came from the 7% price increase scenario, which we selected as the most realistic benchmark because similar price changes have occurred in states that restrict alcohol sales to state-controlled outlets. The lower and upper uncertainty interval (UI) estimates came from the most conservative (no price increase) and least conservative (price increased until a consumption target reduction of 5% is met) scenarios, respectively. For the simulation, a triangular distribution for the reduction in consumption parameter was used in each state based on estimates from the 3 modeled scenarios. For example, in Oregon, Diaz et al.^12^ estimated a 3.3%, 1.9%, and 5.2% reduction in cigarette sales for the 7% price increase, no price increase, and target reduction of 5% scenarios, respectively. Therefore, for the simulation in this study, a triangular distribution was used where 3.3% was considered the most likely estimate, and 1.9% and 5.2% were selected as the lowest and highest possible estimates, respectively.

Next, informed by studies on the effects of raising price on cigarette sales,^19,20^ we assumed that half of the reduction in cigarette sales each year would be due to reduction in smoking prevalence and the other half would be due to individuals who smoke reducing their consumption. There is no published evidence identifying the proportion of the reduction in smoking prevalence as a result of the policy that would be due to changes in initiation, relapse, and quitting. The population affected by the policy at risk for initiation and relapse is likely smaller than the population of current smokers. Therefore, we assumed that the reduction in smoking prevalence would be 80% driven by individuals who quit smoking, with 10% of the reduction instead due to individuals who no longer initiate smoking and 10% due to individuals who no longer relapse. In addition, for individuals who do not initiate cigarette smoking as a result of the policy, we assumed that there would be no tobacco-related mortality benefit over a 20-year period, as compared to if they had initiated. Most individuals first try smoking by early adulthood (<18 years),^21^ and the likelihood of tobacco-related death for both never and current smokers before 40 years is low.^22^

To determine the change in the likelihood of death due to individuals who quit smoking or no longer relapse as a result of the policy, we used data on mortality risk for former smokers, as compared to current smokers, by years since an individual quit (<2 years, 2-4 years, 5-9 years, 10-19 years, and 20+ years).^23^ Finally, informed by a systematic review that found no significant relationship between reduced consumption and risk of all-cause mortality,^24^ we assumed that there is no mortality benefit for individuals who reduce their consumption in cigarette use due to the policy.

Model values and data sources are provided in Table 1. We used the following equation to estimate the mortality reduction over 20 years: A^*^(B^*^C)^*^$\sum_{k=1}^5{W}_k{D}_k$*.* Here, *A* represents the number of annual deaths due to cigarette smoking in each state; *B* is the state-specific percentage annual reduction in sales due to the policy, *C* is the proportion reduction in sales attributable to reduced smoking prevalence resulting from individuals who quit or no longer relapse (0.45 [0.90/2] in the main models); *D* is the mortality risk ratio for former smokers who quit 1-20 years ago compared with current smokers; and *W* denotes a weighting factor corresponding to the number of years represented within each mortality risk interval (eg, for the mortality risk estimate for 2-4 years, *w* = 3). To incorporate uncertainty into the analysis, we used Monte Carlo simulation with repeated random sampling. In each simulation iteration, values for all uncertain model parameters were jointly sampled from their respective probability distributions (see Appendix), and the model was evaluated deterministically using these sampled values. This procedure was repeated 100 000 times to generate an empirical distribution of mortality outcomes in each state.

**Table 1:** Model values, data sources, and estimates of policy impact on tobacco-related mortality.

State	A. Deaths from smoking	B. Percentage annual reduction in sales due to the policy	C. Proportion of reduction in sales due to reduction in prevalence	D. Mortality risk ratio for former smokers who quit 1-20 years ago vs. current smokers		Mortality reduction over 20-year period (95% UI)
**Oregon**	6586 (5603, 7738)	3.3% (1.9%, 5.2%)		20+ years	1-0.54 (0.51, 0.58)	366 (169, 652)
				10-19 years	1-0.77 (0.72, 0.82)	
				5-9 years	1-0.83 (0.77, 0.91)	
				2-4 years	1-1.02 (0.93, 1.13)	
				1 year	1-1.07 (0.95, 1.21)	
**Pennsylvania**	24 673 (20 934, 28 903)	3.8% (2.4%, 5.8%)		20+ years	1-0.54 (0.51, 0.58)	1585 (735, 2773)
				10-19 years	1-0.77 (0.72, 0.82)	
				5-9 years	1-0.83 (0.77, 0.91)	
			45%	2-4 years	1-1.02 (0.93, 1.13)	
			(22.5%, 67.5%)	1 year	1-1.07 (0.95, 1.21)	
**Vermont**	1143 (1021, 1290)	3.8% (2.4%, 5.7%)		20+ years	1-0.54 (0.51, 0.58)	74 (34, 127)
				10-19 years	1-0.77 (0.72, 0.82)	
				5-9 years	1-0.83 (0.77, 0.91)	
				2-4 years	1-1.02 (0.93, 1.13)	
				1 year	1-1.07 (0.95, 1.21)	
**Virginia**	13 038 (11 047, 15 115)	4.8% (3.4%, 6.7%)		20+ years	1-0.54 (0.51, 0.58)	1064 (501, 1.806)
				10-19 years	1-0.77 (0.72, 0.82)	
				5-9 years	1-0.83 (0.77, 0.91)	
				2-4 years	1-1.02 (0.93, 1.13)	
				1 year	1-1.07 (0.95, 1.21)	

We used the following equation to estimate mortality reduction over 20 years: A^*^(B^*^C)^*^$\sum_{k=1}^5{W}_k{D}_k$*,* where *w* refers to a weight reflecting the number of years in the mortality risk estimate grouping (eg, for the mortality risk estimate for 2-4 years, *w* = 3). Estimates on annual deaths due to cigarette smoking came from the Global Burden of Disease Study.^17^ Estimates of the percentage annual reduction in sales came from Diaz et al.^12^ Estimates of the proportion of reduction in sales due to individuals quitting or no longer relapsing was informed by studies on the effects of raising price on cigarette sales,^19,20^ along with assumptions made by the research team. Estimates of the mortality risk ratio for former smokers came from Thun et al.^23^

We also conducted 2 sensitivity analyses. In 1 we reduced the price elasticity of demand from -0.4 to a more conservative estimate of -0.2. In the other, the price elasticity of demand remained at -0.4, but we lowered the overall percentage reduction in smoking prevalence due to cessation (quitting and no longer relapsing) from 90% to 80%. We assumed that the reduction in smoking prevalence would be 70% driven by individuals who quit smoking, with 20% of the reduction due to individuals who no longer initiate smoking and 10% due to individuals who no longer relapse.

## Results

In Oregon, Pennsylvania, Vermont, and Virginia, the Global Burden of Disease study estimates that 6586 (95% CI = 5603, 7738), 24 673 (95% CI = 20934, 28 903), 1143 (95% CI = 1021, 1290), and 13 038 (95% CI = 11047, 15 115) individuals die from cigarette smoking each year, respectively. Based on calculations by Diaz et al.,^12^ we assumed cigarette consumption decreased by 3.3% (1.9%, 5.2%), 3.8% (2.4%, 5.8%), 3.8% (2.4%, 5.7%), and 4.8% (3.4%, 6.7%) in Oregon, Pennsylvania, Vermont, and Virginia, respectively. In each state, half of this reduction was expected to be due to individuals ceasing use of tobacco products (quitting or no longer relapsing) or not initiating smoking. Based on these assumptions, we estimated that over a 20-year period 366 (95% UI: 169, 652), 1585 (95% UI: 735, 2773), 74 (95% UI: 34, 127), and 1064 (95% UI: 501, 1806) deaths due to cigarette smoking would be avoided in Oregon, Pennsylvania, Vermont, and Virginia, respectively. Results from sensitivity analyses with more conservative model assumptions were in expected directions (Appendix Tables 1 and 2). Across states, it took 5 years or more for mortality benefits to be experienced after policy implementation (Figure 1).

**Figure 1:**
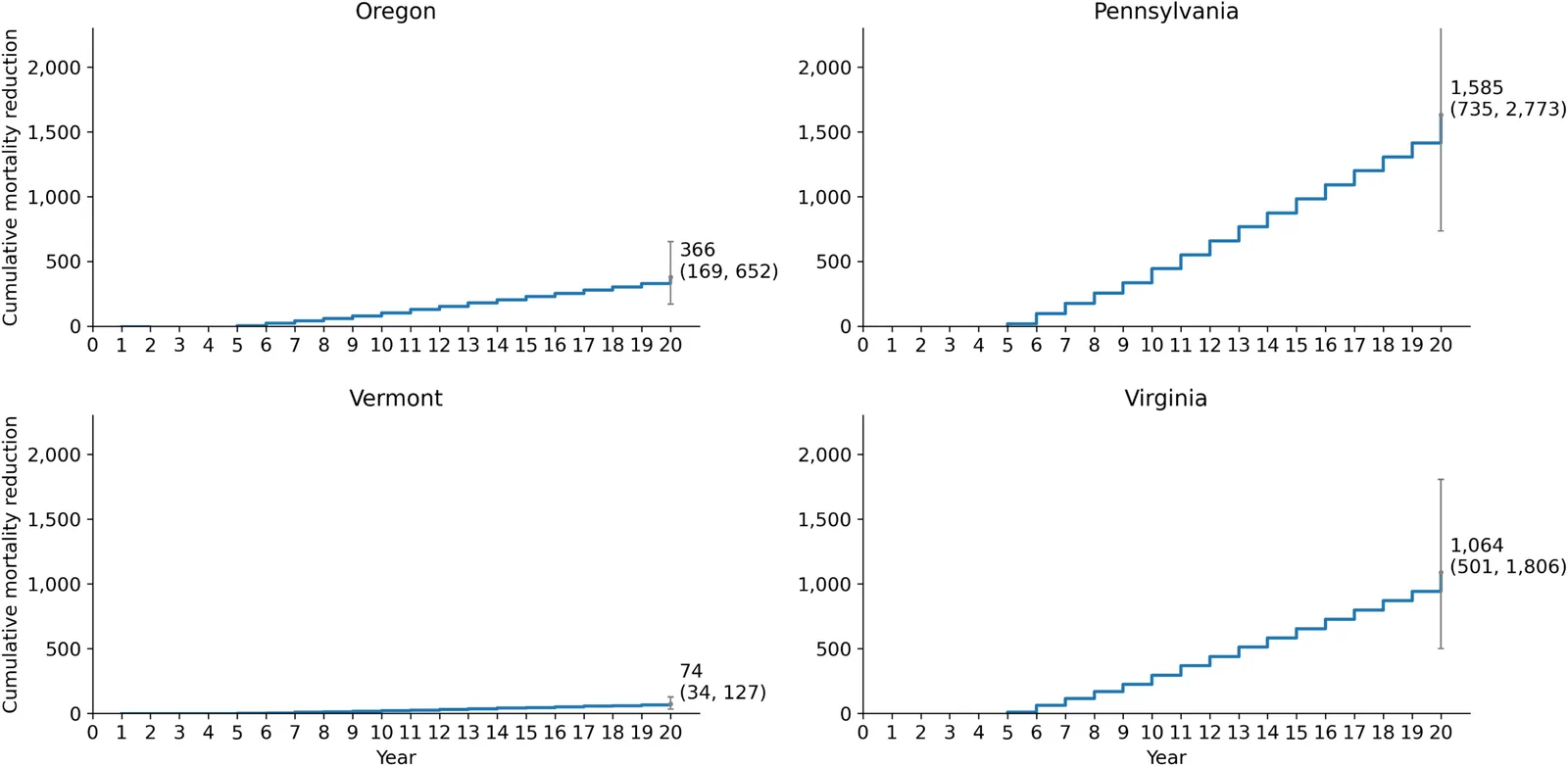
Cumulative reduction in mortality in Oregon, Pennsylvania, Vermont, and Virginia. *Notes*. The figure presents the estimated cumulative reduction in mortality over a 20-year period as a result of transitioning cigarette sales to state-controlled outlets in each state.

## Discussion

In each of the 4 states examined, we found that restricting cigarette sales to state-controlled outlets would substantially reduce cigarette-related deaths over a 20-year period. Despite initial differences in cigarette prices and tobacco retailer locations, the percentage of deaths averted as a result of the policy was fairly consistent across states. States with a higher baseline burden of tobacco-related disease experienced greater absolute reductions in mortality. It took several years for policy impacts on mortality to be realized because mortality risk does not decline until at least 5 years after an individual has quit smoking.^23^

Prior simulation modeling research has estimated the impacts of substantially increasing cigarette taxes, another endgame strategy, on smoking-attributable mortality in US states.^25^ Compared to restricting cigarette sales to state-controlled outlets, increasing cigarette taxes by $1.00 in 2023 is estimated to result in a larger reduction in smoking-attributable mortality in Oregon (2110 vs 366 deaths), Pennsylvania (4780 vs 1585 deaths), Vermont (221 vs 74 deaths), and Virginia (4780 vs 1064 deaths).^25^ Although increasing cigarette taxes may have a greater impact, findings from this study suggest both policies are strong tobacco control interventions. For certain states, restricting cigarette sales to state-controlled outlets may have greater public or political support than raising taxes. Seventeen states, including Pennsylvania, Virginia, and Vermont, already regulate the sale of alcoholic beverages and may be particularly amenable to extending such regulations to tobacco products.^15^ All non-preempted states, however, could pursue this tobacco control strategy because the 2009 US Family Smoking Prevention and Tobacco Act permits local jurisdictions to adopt laws that regulate the sale and distribution of tobacco products.^26^

This study has some limitations. Although we used reliable estimates about deaths due to cigarette smoking and reasonable assumptions from the literature, results from this study are dependent on model assumptions. To understand the potential range of policy impacts, we considered different plausible scenarios where states would maintain cigarette pack prices at their current levels or enact modest increases. In addition, we assumed the policy impact on consumption would be stable in each year over time. If states implemented larger increases on cigarette pack prices than in the policy scenarios evaluated, we expect impacts on cigarette smoking, and in turn, mortality, would be greater. We also used conservative assumptions that there would be no reduction in mortality over a 20-year period among individuals who no longer initiate smoking and among individuals who reduce their consumption as a result of the policy. Finally, this study focused on 4 states for which we had data on the expected change in cigarette consumption as a result of the policy. Although the model values in this study are state-specific, the projected policy impact over 20 years ranged from 5.5% to 8% of the annual cigarette-attributable deaths across a diverse set of states. Therefore, other states could approximate the policy’s 20-year mortality impact by multiplying this same range (5.5%-8%) by the state’s annual number of cigarette-attributable deaths.

Overall, findings from this study suggest that transitioning cigarette sales to state-controlled outlets is a strong tobacco control strategy that may reduce tobacco-related mortality. Public health practitioners and policymakers can use estimates from this study to inform the selection of this potential endgame policy in their state.

## Supplementary Material

ntag084_Appendix_TTS_Mortality

## Data Availability

The data used in the present study are publicly available. The analytic code can be accessed on the University of North Carolina at Chapel Hill Dataverse, an open-source digital repository (https://dataverse.unc.edu/dataverse/restrictingcigarettesales).
