# Supplementary material for: The impact of restricting US cigarette sales to state-controlled outlets on tobacco-related mortality
Source: Nicotine Tob Res. 2026 Apr 21;28(11):1967–71. doi: 10.1093/ntr/ntag084 (PMC13687905; doi:10.1093/ntr/ntag084)
Supplement: Supplementary file 1 [file ntag084_appendix_tts_mortality.docx]

**Appendix**

**Probability Distributions Used for the Monte Carlo Simulation**

To incorporate uncertainty into the analysis for this study, we used Monte Carlo simulation with repeated random sampling. In each simulation iteration, values for all uncertain model parameters were jointly sampled from their respective probability distributions, and the model was evaluated deterministically using these sampled values. This procedure was repeated 100,000 times to generate an empirical distribution of smoking-attributable mortality outcomes.

Deaths from smoking were sampled from a log-normal distribution based on published point estimates and confidence intervals. The percentage annual reduction in cigarette sales due to the policy was sampled from triangular distributions. The point estimate for the percentage annual reduction in cigarette sales was taken from the 7% price increase scenario in Diaz et al.,^12^ which we selected as the most realistic benchmark because similar price changes have occurred in states that restrict alcohol sales to state-controlled outlets. The lower and upper uncertainty interval estimates came from the most conservative (no price increase) and least conservative (price increased until a consumption target reduction of 5% is met) scenarios, respectively.^12^ The proportion of the reduction in cigarette sales attributable to reductions in smoking prevalence was sampled from a logistic distribution bounded between 0 and 1. Mortality risk ratios for former smokers who quit 1–20 years earlier compared with current smokers were sampled from log-normal distributions based on published relative risk estimates and modeled across time since cessation. The resulting distribution of outcomes across all simulation runs was used to calculate point estimates (means) and 95% uncertainty intervals, defined by the 2.5th and 97.5th percentiles of the simulated values.

| **Appendix Table 1**. Sensitivity Analysis using -0.2 Price Elasticity of Demand | | | | | | | |
| --- | --- | --- | --- | --- | --- | --- | --- |
| State | A. Deaths from smoking | B. Percentage annual reduction in sales due to the policy | C. Proportion of reduction in sales due to reduction in prevalence | D. Mortality risk ratio for former smokers who quit 1-20 years ago vs. current smokers | | Mortality reduction over 20-year period (95% UI) | |
| Oregon | 6586  (5603, 7,738) | 1.6%  (0.9%, 2.6%) |  | 20+ years | 1 – 0.54 (0.51, 0.58) | | 160  (70, 298) |
|  |  |  |  | 10-19 years | 1 – 0.77 (0.72, 0.82) | |  |
|  |  |  |  | 5-9 years | 1 – 0.83 (0.77, 0.91) | |  |
|  |  |  |  | 2-4 years | 1 – 1.02 (0.93, 1.13) | |  |
|  |  |  |  | 1 year | 1 – 1.07 (0.95, 1.21) | |  |
| Pennsylvania | 24,673  (20,934, 28,903) | 1.9%  (1.2%, 2.9%) |  | 20+ years | 1 – 0.54 (0.51, 0.58) | | 714  (314, 1,299) |
|  |  |  |  | 10-19 years | 1 – 0.77 (0.72, 0.82) | |  |
|  |  |  |  | 5-9 years | 1 – 0.83 (0.77, 0.91) | |  |
|  |  |  | 45% | 2-4 years | 1 – 1.02 (0.93, 1.13) | |  |
|  |  |  | (22.5%, 67.5%) | 1 year | 1 – 1.07 (0.95, 1.21) | |  |
| Vermont | 1,143  (1,021, 1,290) | 1.9%  (1.2%, 2.9%) |  | 20+ years | 1 – 0.54 (0.51, 0.58) | | 33  (15, 60) |
|  |  |  |  | 10-19 years | 1 – 0.77 (0.72, 0.82) | |  |
|  |  |  |  | 5-9 years | 1 – 0.83 (0.77, 0.91) | |  |
|  |  |  |  | 2-4 years | 1 – 1.02 (0.93, 1.13) | |  |
|  |  |  |  | 1 year | 1 – 1.07 (0.95, 1.21) | |  |
| Virginia | 13,038  (11,047, 15,115) | 2.4%  (1.7%, 3.4%) |  | 20+ years | 1 – 0.54 (0.51, 0.58) | | 478  (215, 848) |
|  |  |  |  | 10-19 years | 1 – 0.77 (0.72, 0.82) | |  |
|  |  |  |  | 5-9 years | 1 – 0.83 (0.77, 0.91) | |  |
|  |  |  |  | 2-4 years | 1 – 1.02 (0.93, 1.13) | |  |
|  |  |  |  | 1 year | 1 – 1.07 (0.95, 1.21) | |  |
| *Notes.* We used the following equation to estimate the mortality reduction over 20 years: A*(B*C)*$\sum_{k=1}^{5} W_{k}D_{k}$*,* where *w* refers to a weight reflecting the number of years in the mortality risk estimate grouping (e.g., for the mortality risk estimate for 2-4 years, *w* = 3). For this sensitivity analysis, we used a -0.2 price elasticity of demand. **Appendix Table 2**. Sensitivity Analysis with a Reduced Estimate of the Proportion Reduction in Sales due to a Change in Prevalence \| \| \| \| \| \| \|; State \| A. Deaths from smoking \| B. Percentage annual reduction in sales due to the policy \| C. Proportion of reduction in sales due to reduction in prevalence \| D. Mortality risk ratio for former smokers who quit 1-20 years ago vs. current smokers \| \| Mortality reduction over 20-year period (95% UI) \|; Oregon \| 6586 (5603, 7,738) \| 3.3% (1.9%, 5.2%) \| \| 20+ years \| 1 – 0.54 (0.51, 0.58) \| \| 160 (70, 298); \| \| \| \| 10-19 years \| 1 – 0.77 (0.72, 0.82) \| \|; \| \| \| \| 5-9 years \| 1 – 0.83 (0.77, 0.91) \| \|; \| \| \| \| 2-4 years \| 1 – 1.02 (0.93, 1.13) \| \|; \| \| \| \| 1 year \| 1 – 1.07 (0.95, 1.21) \| \|; Pennsylvania \| 24,673 (20,934, 28,903) \| 3.8% (2.4%, 5.8%) \| \| 20+ years \| 1 – 0.54 (0.51, 0.58) \| \| 714 (314, 1,299); \| \| \| \| 10-19 years \| 1 – 0.77 (0.72, 0.82) \| \|; \| \| \| \| 5-9 years \| 1 – 0.83 (0.77, 0.91) \| \|; \| \| \| 40% \| 2-4 years \| 1 – 1.02 (0.93, 1.13) \| \|; \| \| \| (20%, 60%) \| 1 year \| 1 – 1.07 (0.95, 1.21) \| \|; Vermont \| 1,143 (1,021, 1,290) \| 3.8% (2.4%, 5.7%) \| \| 20+ years \| 1 – 0.54 (0.51, 0.58) \| \| 33 (15, 60); \| \| \| \| 10-19 years \| 1 – 0.77 (0.72, 0.82) \| \|; \| \| \| \| 5-9 years \| 1 – 0.83 (0.77, 0.91) \| \|; \| \| \| \| 2-4 years \| 1 – 1.02 (0.93, 1.13) \| \|; \| \| \| \| 1 year \| 1 – 1.07 (0.95, 1.21) \| \|; Virginia \| 13,038 (11,047, 15,115) \| 4.8% (3.4%, 6.7%) \| \| 20+ years \| 1 – 0.54 (0.51, 0.58) \| \| 478 (215, 848); \| \| \| \| 10-19 years \| 1 – 0.77 (0.72, 0.82) \| \|; \| \| \| \| 5-9 years \| 1 – 0.83 (0.77, 0.91) \| \|; \| \| \| \| 2-4 years \| 1 – 1.02 (0.93, 1.13) \| \|; \| \| \| \| 1 year \| 1 – 1.07 (0.95, 1.21) \| \|; *Notes.* We used the following equation to estimate the mortality reduction over 20 years: A*(B*C)*$\sum_{k=1}^{5} W_{k}D_{k}$*,* where *w* refers to a weight reflecting the number of years in the mortality risk estimate grouping (e.g., for the mortality risk estimate for 2-4 years, *w* = 3). For this sensitivity analysis, the price elasticity of demand remained at -0.4, but we lowered the overall percentage reduction in sales due to individuals quitting and no longer relapsing from 45% (main analysis) to 40%. \| \| \| \| \| \| \| | | | | | | | |
